# Relationship between Pain, Fear of Falling and Physical Performance in Older People Residents in Long-Stay Institutions: A Cross-Sectional Study

**DOI:** 10.3390/ijerph191912014

**Published:** 2022-09-22

**Authors:** Sabrina Gomes Fernandes, Weslley Barbosa Sales, Diego Villar Tavares, Dayanna da Silva Pereira, Patrícia Vidal de Negreiros Nóbrega, Cristina Marques de Almeida Holanda, Alvaro Campos Cavalcanti Maciel

**Affiliations:** Physiotherapy Department, Federal University of Rio Grande do Norte, Natal 59078-970, Brazil

**Keywords:** aging, aged, pain, functional performance, accidents due to falls, institutionalized older people

## Abstract

**Introduction:** To analyze the relationship between pain, the fear of falling and functional performance in older people living in a long-stay institution (LSI) in the interior of northeastern Brazil. **Methods:** A cross-sectional study was conducted with 133 older residents in an LSI in the State of Paraíba. The instruments used for data collection were the Geriatric Pain Measure (GPM), the Falls Efficacy Scale-International (FES-I) and the Short Physical Performance Battery (SPPB). **Results:** Pain was reported by 57.5% of those evaluated, 48% being classified as chronic pain and presenting an average of 25.2 in the GPM. As for physical performance, assessed using the SPPB, the 133 older residents showed moderate to poor performance, with an average of 6.43 (±2.96) on the scale. By correlating the adjusted GPM values with the FES-I, a weak and statistically significant positive correlation was obtained (ρ = 0.31: *p* < 0.001). **Conclusions:** It can be concluded that those who reported pain had a worse performance in the applied tests, in addition to having higher scores on the scale referring to a fear of falling.

## 1. Introduction

Population aging is occurring significantly around the world, with a consequent increase in the number of people over 60 years of age [1]. Due to this, the emergence of new challenges for health services is an inevitable fact, since the anatomical changes themselves increase the functional impairment of older people, causing a need for more specialized care [2].

Given this situation, the process of institutionalizing the older population appears as an alternative for families that do not have the physical, financial and psychological conditions to provide full-time care to their older people, opting for long-stay institutions (LSI) [3].

However, in this new environment, autonomy becomes more restricted, due to situations that can trigger a loss of identity, decreased self-esteem and social isolation [4]. Likewise, it can promote decreased mobility and functional capacity, an increased fear of falling and the occurrence of pain processes, particularly those related to chronic degenerative diseases [5]. It is estimated that more than 60% of older people living in LSIs have at least one report of pain throughout the year [6].

Regarding the possible relationship between pain and the fear of falling, it is assumed that pain can negatively influence muscle strength, motor coordination, body sway and stability, the individual’s proprioception and even his level of cognitive function, all of which are directly linked to this feeling of fear [6].

Similarly, limitations in the functional performance of older people, caused by pain, have a multidimensional character, but it is still a challenge to elucidate the mechanisms involved more precisely [7]. To date, the available empirical evidence demonstrates that pain is one of the most potent predictors of disability and falls in geriatric populations, both in the community and in long-term care facilities [8].

It is suggestive that the pain complaint of the older population deserves greater care and attention from professionals linked to geriatric rehabilitation. Daily discomfort can accompany not only the difficulties in performing basic Activities of Daily Living (ADL), but, above all, it can constitute a factor for the increase of the fear of falling, bringing negative and even fatal consequences for the older population and giving rise to a considered increase in public spending for the rehabilitation of this older person [7,8].

The relationship between pain [9] and the fear of falling with physical performance is already well established in the literature [10,11,12]; however, these investigations were viewed separately, that is, to the best of our knowledge, no other study has analyzed the association between these three variables together, especially in a population residing in an LSI. One hypothesis for this is that both pain and the fear of falling lead to a non-performance of daily activities, which can lead to a series of complications, especially in the functional capacity, independence and autonomy of these elderly people. Therefore, investigating the association between these variables helps us to understand whether the presence of pain and the fear of falling will negatively influence the physical performance of this public.

From this perspective, proposing a study that encompasses the dimensions of pain, the fear of falling and functional performance can help health professionals, especially geriatric physical therapists, in the elaboration of public policies to reduce adverse outcomes, the elaboration of specific evaluation protocols and treatments, and ensuring that preventive and rehabilitative measures are taken to ensure a better quality of life, autonomy and independence for this population group which is so susceptible to adverse health outcomes [12].

In view of the above, the objective of this study was to analyze the relationship between pain, the fear of falling and functional performance in older people living in an LSI located in the interior of northeastern Brazil. This region was chosen because it is a low-income place, with high rates of social inequality that affect individuals’ access to quality health care, with a direct impact on the health and functionality conditions of older people.

## 2. Materials and Methods

### 2.1. Study Design

This is a cross-sectional study conducted with older people living in an LSI in the state of Paraíba, from May to July 2016. The study design was based on recommendations from the Strengthening the Reporting of Observational Studies in Epidemiology (STROBE) for observational studies [13].

### 2.2. Participants

The population of this research consisted of older people living in an LSI in the first and second healthcare macro-regions of the State of Paraíba, polarized by the municipalities of João Pessoa and Campina Grande. Initially, a survey was conducted with the State Department for Human Development, where 981 older people were living in 25 LSIs.

The sample calculation was performed using the website (https://calculareconverter.com.br/calculo-amostral/, (accessed on 10 January 2015)), the estimated prevalence of the studied outcome was observed and the following statistical parameters were adopted: maximum statistical errors of 5.0% for type I and 20.0% for type II, study power of 80.0%, a finite population size of 828 subjects and an estimated disease prevalence of 30.0%; 15.0% was added to the value to compensate for the occurrence of some selection bias. As a result, we found a representative sample of the population of 133 subjects allocated in a simple random probabilistic way.

### 2.3. Eligibility Criteria

The sample of the present study consisted of older people (≥60 years old) residing in an LSI, without a history of cancer that could refer to cancer pain, without any clinical or functional situation that made it impossible to respond coherently to the data collection instruments and that did not present severe cognitive disorders, according to the Mini-Mental State Examination (MMSE), which for this research, the cut-off point of 17 was adopted, defined by the methodology used by the *Rede de Estudos de Fragilidade de Idosos Brasileiros* project [14,15,16].

Those older people who decided to withdraw from the research during data collection, those who had unfavorable conditions for continuing the study and those who did not complete the chronic pain assessment or physical performance tests during the assessment were excluded.

**Figure 1:**
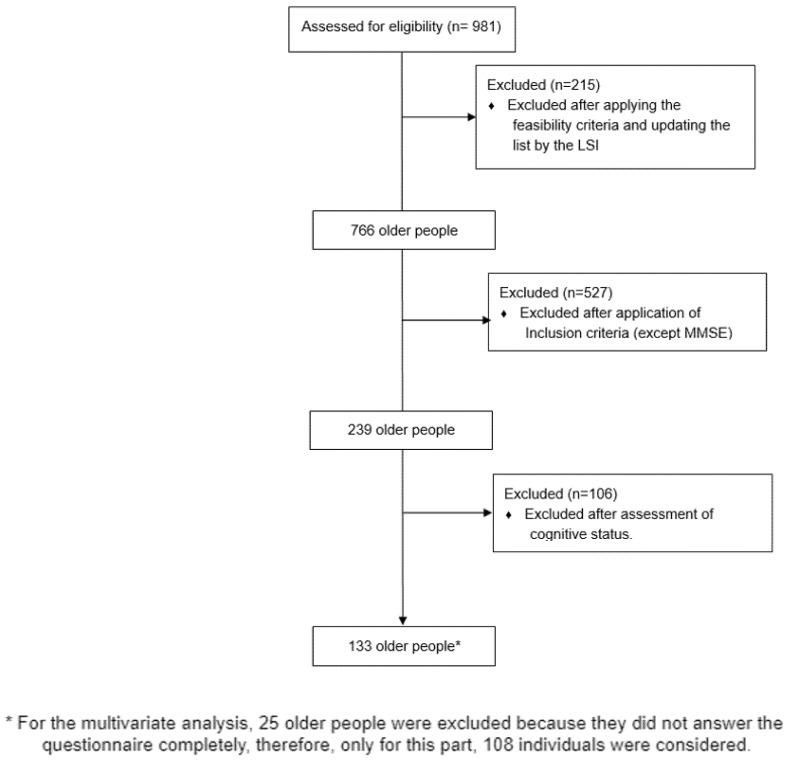
Sample selection flowchart for the study.

Altogether, 133 subjects remained in the study; however, for the analysis regarding the association between the fear of falling and physical performance, 25 older people were unable to complete the questionnaire, thus, only 108 subjects were considered. There are further details in Figure 1.

### 2.4. Procedures

All participants were assessed by trained interviewers using the standardized questionnaire described below:

#### 2.4.1. Sociodemographic Variables

Information on gender and age (in years) was collected. For marital status, responses were categorized as married, single, divorced or widowed; in the assessment of schooling, it was asked how many years of complete education the older person had and the answers were categorized as illiteracy, low schooling or high schooling.

#### 2.4.2. Body Mass Index (BMI)

BMI was measured using a digital scale, with an accuracy of 0.1 kg, and a stadiometer, with an accuracy of 0.1 cm. From the collection of weight and height information, the BMI was calculated using the standard method (BMI = weight/height^2^) and classified as: malnutrition (<22 kg/m^2^), eutrophy (22–27 kg/m^2^) or obesity (≥27 kg/m^2^) [17].

#### 2.4.3. Use of Psychotropic and/or Hypnotic Drugs

It was asked if the older person used any type of psychotropic and/or hypnotic drugs. For this variable, responses were dichotomized into yes or no [17].

#### 2.4.4. Perceived Health

The subject was asked to perform a subjective assessment of their health, in which they were asked, *“In general, would you say your health is?”*, with the answers dichotomized into “satisfactory” or “unsatisfactory” [17].

#### 2.4.5. Depression

The *Geriatric Depression Scale (GDS-15*) [18] was used to assess depressive symptoms. This scale is often used to screen for depressive disorders [19]. It is a validated instrument [20], culturally adapted and translated to Brazil [21] and that has already been used in LSIs [22].

#### 2.4.6. Pain

Initially, they were asked about the time of onset of pain, and later, classified as acute, if present for up to six months, or chronic, if the referred duration was longer than six months [8].

For pain assessment, the *Geriatric Pain Measure* (GPM) questionnaire was also used, which was developed to allow an assessment of pain in its various domains. Composed of 22 items with dichotomous answers, and two items composed of a scale from 0 to 10, the total score is obtained by adding the “yes” answers to the dichotomous items with the numerical answers to the other questions, resulting in a total score. Thus, the total score can range from 0 to 42, with zero meaning no pain and 42 meaning the worst pain. The GPM stands out for assessing the multiple dimensions of pain, such as intensity, “disengagement”, pain when walking, pain in vigorous activities and pain in other activities [23].

#### 2.4.7. Fear of Falling

To assess the fear of falling, the *Falls Efficacy Scale-International* (FES-I) was used [24]. The FES-I presents questions about the concern of the possibility of falling when performing 16 activities, with respective scores from one to four. The overall score can range from 16 (no concern) to 64 (extreme concern). Scores between 16 and 19 points are indicative of a low concern with fear of falling, scores between 20 and 27 points indicate a moderate concern and scores between 28 and 64 points are suggestive of a high concern with fear of falling [25].

#### 2.4.8. Short Physical Performance Battery

**Figure 2:**
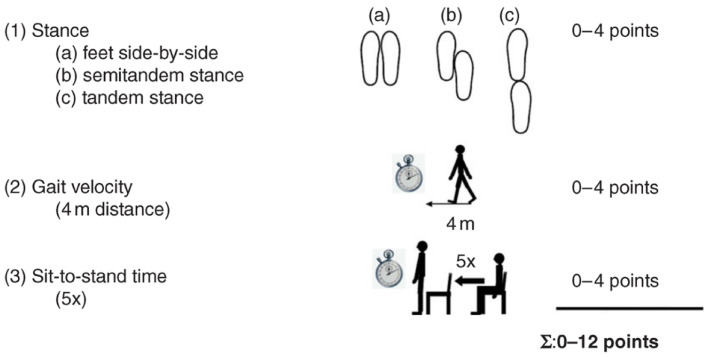
Illustrative image of the activities carried out at SPPB. (1) Balance test: (**a**) feet side-by-side position; (**b**) semitandem position; (**c**) tandem position.

This instrument is effective in assessing the physical performance of the older population. It consists of three tests that are evaluated in sequence: (1) static balance while standing; (2) the walking speed at usual pace, measured twice on a given round trip; and, indirectly, (3) the muscular strength of the lower limbs, through the movement of getting up and sitting down in the chair five consecutive times without the help of the upper limbs (Figure 2). The total score is obtained by adding the scores of each test, ranging from zero (worst performance) to twelve points (best performance). For the analysis of the data in this article, information on the total SPPB score was considered, as well as the values found for each test, separately.

### 2.5. Ethical Aspects

The research project was submitted for evaluation by the Research Ethics Committee of the Federal University of Rio Grande do Norte (CEP/UFRN), obtaining approval and a favorable opinion for its realization (opinion No. 834,782; date: 26 September 2014). All research participants signed the Free and Informed Consent Term, according to the recommendations in Resolution No. 466, of 12 December 2012, of the National Health Council/MS and the Declaration of Helsinki.

### 2.6. Data Analysis

Data analysis was performed using the SPSS statistical program (*Statistical Package for the Social Sciences*, IBM®, Armonk, NY, USA), version 20.0. Initially, data normality was verified using the Kolmogorov–Smirnov test. Descriptive statistics were performed using distribution measures (mean and standard deviation). For inferential statistics, Student’s *t*-test and Spearman’s correlation test were used.

For the last step of the statistical analysis, a multiple linear regression was performed to verify the relationship between pain and outcome measures (fear of falling and physical performance). The exit criterion for all variables introduced in the model was *p* < 0.20. In all statistical analysis, a confidence interval (CI) of 95% and *p* < 0.05 was considered.

## 3. Results

Data from 133 older people with a mean age of 78.8 (±7.62) and 3.80 (±3.49) years of institutionalization were analyzed. There was a predominance of females (65.4%), who were single (43.2%) and had a low education (59.2% with only a basic education and 23.8% were illiterate). Regarding nutritional status, 45.4% of the subjects evaluated were classified as eutrophic (45.4%), with a mean body mass index of 24.88 kg/m^2^ (±5.13). Regarding the MMSE and GDS-15 scores, the institutionalized older people obtained means of 21.5 (±3.5) and 5.7 (±3.2) points, respectively. Variables such as marital status, education, nutritional status and use of psychotropic and hypnotic drugs have missing values (1–3 people); however, the absence of these values does not affect the statistical analysis. The other data are present in Table 1.

Table 2 shows the characterization of the dependent and independent variables of the study. Considering the 133 subjects, it was seen that pain was reported by 57.5% of those evaluated, with 48% being classified as in chronic pain and presenting an average of 25.2 in the GPM. Regarding the fear of falling scale (FES-I), an average of 34.46 (± 11.6) points was found. When categorizing the FES-I, it was also observed that, of the 108 interviewees evaluated with this scale, 69.4% had a score higher than 28 points.

As for physical performance, assessed using the SPPB, in general, the older population showed moderate to poor performance, with an average of 6.43 (±2.96) in the total score of the scale, which ranges from 0 to 12 points. The other results for the tests evaluated are observed in Table 2.

When correlating the performance in SPPB and the FES-I with the presence of pain and the GPM score, in general, weak correlations were observed between the analyzed variables. For the gait speed and total score tests, this correlation was negative, showing that the higher the score in the pain items, the lower the speed performed and the worse the total performance.

In relation to the chair-rising test, the correlation was positive, demonstrating that the higher the score in the pain items, the longer the time that was spent to perform the test. For the FES-I outcome, there was a statistically significant difference in the variables of the presence of pain (*p* < 0.001) and chronic pain (*p* = 0.001). In addition, when the overall GPM score was correlated with the FES-I, a positive and weak, but statistically significant, correlation was found. All data are presented in Table 3.

The multivariate analysis of physical performance is presented in Table 4. Analysis models were developed for each SPPB component and its total score was adjusted by the covariates. For balance, it was seen that only age and the use of hypnotic drugs remained in the model, and these had a negative influence on the scores obtained by the older people (**ß** = −0.045; *p*-value = 0.01 and **ß** −0.64; *p*-value = 0.03, respectively).

As for the assessment of gait speed, the result was that as each year increases in age, there is a decrease of 0.01 m/s in gait speed (*p* = 0.01). In addition, it was also found that an older person with acute pain showed a decrease of 0.23 m/s in gait speed (*p* = 0.03), a considerably high value in relation to the average speed of the general sample (0.73 m/s ± 0.35). For the test to get up from the chair, it was seen that a subject with acute pain had an increase of 3.7 s in the time needed to perform the test, which demonstrates greater slowness in movements (*p* = 0.04).

Finally, for the multivariate analysis of the SPPB total score, it was found that for each year increased in age, there was a loss of 0.1 points in performance (*p* = 0.01) and that older people with acute pain showed a decrease of 1.9 points in the total score of SPPB, expressive loss, considering the average performance of the general sample (6.43 ± 2.96). Chronic pain did not appear as a limiting factor in the performance of the older people in this analysis.

A multiple linear regression analysis was also performed to verify the relationship between pain and the FES-I. Three models were constructed and adjusted each for the presence of pain in general (dichotomous), pain intensity by GPM and the presence of chronic pain, respectively (Table 5).

In all models, the variables of gender, use of psychotropic drugs, perceived health, age and GDS score were included. In the first regression model for the presence of pain, the statistically significant variables showed that the presence of pain in general causes an increase of 5.47 points in the FES-I, the fact that the subject is male causes a decrease of 4.6 points. As for the older person who did not use psychotropic drugs, there was a reduction of 4.7 points in the FES-I, and in relation to the GDS score, it was seen that every 1 point in the GDS increases by 1.06 in the FES-I.

In the regression model, adjusted by the categorical GPM, it was found that the subjects with severe pain had an increase of approximately 7 points in the FES-I, when compared to those with mild pain. The male subjects, on the other hand, decreased by 5.06 points in the FES-I, those who did not use psychotropic drugs showed a reduction of 4.44 points the FES-I and finally, for every 1 point on the GDS, there was an increase of 1.05 points in the FES-I.

In the third model, adjusted for chronic pain, it was found that the presence of this type of pain resulted in an increase of 3.82 points on the FES-I scale, male individuals had a decrease of 5.43 points, those who did not who use psychotropic drugs had a decrease of 4.79 points, and for every 1 point on the GDS, there was an increase of 1.04 points in the FES-I.

## 4. Discussion

In this study, the influence of pain on physical performance and the fear of falling in older residents in LSIs was observed, showing that the higher the pain scores, the worse the results of gait speed, balance, time to sit and stand and greater is the fear of falling. The presence of these results can be explained by the characteristics of institutionalized older people [26], in which the majority presented as low education, female, with advanced ages and without a spouse (widowed/single), which are risk factors for the development of disabilities [26,27,28].

The predominance of older people in this study with a low to moderate performance found corroborates the data obtained by Marchon, Cordeiro and Nakano [27] who, when evaluating a sample of 30 institutionalized older people in a follow-up study, found similar results at the baseline and during sample tracking. In the assessment of the fear of falling, the mean score (34.46) was higher than the value found in the scale validation study in Brazil (23.55). This difference can be explained by the fact that institutionalized older people have greater physical and social restrictions and, consequently, a greater fear of falling, with scores higher than 27 points, which is associated with a high concern about falling [26]. These results help to maintain the assertion that institutionalization is still, in most cases, associated with physical dependence.

It is known that functional capacity tends to decrease over the years, especially over 70 years of age. We observed that there were negative correlations between age and functional performance, where for each increase in years lived, the results of performance tests were worse, corroborating studies already carried out [26,27,28].

It should be noted, therefore, that the institutionalized population has a high risk of impairment in functional capacity, both because of its social and clinical characteristics, and because of the institutionalization itself, which imposes on the individual, in most cases, a monotonous, dependent and sedentary routine [28]. The loss of functional capacity is associated with a predisposition to frailty, an increased risk of falls and death, reduced mobility and dependence, generating high-cost care and impairing the quality of life of the older population [29,30].

Regarding pain, there was a prevalence of 57.5% of pain complaints in the population studied, corroborating previous studies [31,32,33]. When verifying the association with performance, it was seen that those who reported pain performed worse on gait speed tests, standing up from a chair and on the SPPB total score. Similar to these data, Pereira et al. [34] showed that older people with pain develop interference with their ADLs, being an important component for the development of disabilities and functional impairment.

Regarding the FES-I, it was seen that the older adults who reported chronic pain were more afraid of falling. Such findings are similar to those found by Lach and Parsons [35], in which positive and significant correlations were observed between the fear of falling and chronic conditions, such as the presence of orthopedic injuries, neurological conditions, the use of psychotropic drugs and the presence of pain. According to Patel et al. [36], this correlation can be justified by the fact that chronic pain is associated with decreased physical activity and a predisposition to situations of disability, contributing to the formation of disuse cycles, causing muscle weakness, loss of balance and change in gait biomechanics, which over time reinforces insecurity and the fear of falling.

In the present study, all pain dimensions showed a correlation, although weak, with worse performances in dynamic tests, in the SPPB total score and in the FES-I score, thus demonstrating a performance deficit for the older people with pain. In the “MOBILIZE Study” carried out in Boston, with the objective of evaluating the influence of chronic pain characteristics on SPPB with a sample of 600 community-dwelling older people, they observed that the best results in all SPPB measures are in pain-free older adults. Regarding the fear of falling, Hubscher et al. [37] observed that older people with pain were 6.4 times more likely to have a low efficacy of falls, that is, they have a higher risk of falling.

In our multivariate analysis, the presence of acute pain and advancing age had an impact on gait speed and on the sit-to-stand test, in addition to promoting a reduction of approximately two points in the total SPPB score. Those who reported severe pain were also seen to have an increase of approximately 7 points in the FES-I.

Therefore, it can be said that our findings can possibly be explained due to the complications of the pain condition, as described by Onder et al. [38], which shows that the presence of pain conditions can generate a reduction in the range of motion or pain and cause a reflex inhibition of the muscular system, resulting in muscle impairment and weakness in physical performance, in addition to leading to a greater risk of falls.

### Strengths and Limitations

The limitations of the present study are highlighted, due to its design (cross-sectional) with a sectional assessment of the pain symptom dependent on self-reporting and the older people’s memory. Pain measurement would have better reproducibility and validity in longitudinal assessments, making it not only a parameter of pain continuity, but increasing the reliability of the information obtained. However, an assessment of the cognitive status of the elderly was carried out, which ensured a greater reliability of the information collected, but limits the generalization of these results for older people with dementia or with cognitive alterations.

In addition, assessing pain is a complex task as it involves both biological and emotional, sociocultural and environmental aspects. To overcome this difficulty, we opted for a multidimensional assessment instrument, translated and validated for the survey population. Another point that should be considered is the scarcity of studies evaluating pain in a multidimensional way, such as using the *Geriatric Pain Measure* (GPM), which has only recently been validated and translated for the elderly and Brazilian population, making it difficult to compare other findings [24].

Another highlight of the study is the use of SPPB as an instrument to assess functional capacity, given that most surveys that relate pain and functional capacity assess the latter through self-reporting. SPPB has already been described as a good identifier of risk subgroups and has advantages over other studies in terms of objectivity, standardization and relationship with multidimensional aspects. In addition, this battery of tests is simple, practical and fast, not requiring much physical space or special material, an important fact for the population studied. In addition, the easy reproducibility and low cost of the assessment instruments used make it possible to carry out similar screening in environments with older people at risk.

There is still a lack of studies that address similar themes in our country, especially in regions such as the northeast of Brazil. In this sense, it is encouraged to carry out research, such as studies of a longitudinal nature, to verify, among other aspects, the chronicity or remission of acute pain in this context, the validity of self-reported pain in these individuals and the importance of pain in the emergence of performance deficits in this population.

## 5. Conclusions

We can conclude that the older people who reported pain present worse results in their physical performance, especially in activities such as walking, balance and sitting to standing; linked to this, these older people obtained a higher score on the fear of falling scale. Therefore, we were able to observe that the presence of pain had a negative impact on the population, associated with a greater risk of adverse health outcomes, such as falls.

Therefore, our findings highlight the need to apply public policies to strengthen the importance of active aging and the role of the geriatric physical therapist in the health team, even in an institutionalized environment, thus avoiding greater commitments to the health of this population group.

## Figures and Tables

**Table 1:** Sample Characterization, Natal-RN, 2016 (*n* = 133).

Variables	Mean or *n*	Standard Deviation (SD) or %
**Age (years)**	78.8	±7.62
**Institutionalization Time (years)**	3.80	±3.49
**Cognitive Function (MMSE)**	21.5	±3.5
**GDS-15 Score**	5.74	±3.24
**BMI (kg/m^2^)**	24.88	±5.13
**Gender**		
Male Female	46 87	34.6% 65.4%
Marital status *		
Married Single Divorced Widower	12 57 25 38	9.1% 43.2% 18.9% 28.8%
Education **		
Illiterate Low education level High schooling	31 77 22	23.8% 59.2% 16.9%
Nutritional Status **		
Malnutrition (<22 kg/m^2^) Eutrophy (22–27 kg/m^2^) Obesity (27 kg/m^2^)	34 59 37	26.2% 45.4% 28.5%
Psychotropic Drug Use *		
Yes No	48 84	36.4% 83.6%
Use of Hypnotic Drugs *		
Yes No	40 92	30.3% 69.7%
**Depression (GDS-15)**		
Yes (>5) No (≤5)	66 67	49.6% 50.4%
**Perceived Health**		
Satisfactory Unsatisfactory	97 36	72.9% 27.1%

Note: * 1 missing value; ** 3 missing value.

**Table 2:** Characterization of pain, fear of falling and physical performance Natal-RN, 2016 (*n* = 133).

Variables	*n* or Mean	% or SD
**Pain in General ^£^**		
Yea No	73 54	57.5% 42.5%
**Acute Pain ^£^**		
Yea No	17 110	13.4% 86.6%
**Chronic Pain ^£^**		
Yea No	61 66	48.0% 52.0%
**Balance Score**	2.73 (±1.44)	-
**Sit and Stand Test-SPPB (s)**	20.27 (±6.41)	-
**Gait Speed Test-SPPB (m/s)**	0.73 (±0.35)	
**SPPB Total Score**	6.43 (±2.96)	-
Physical Performance-SPPB (%) **		
Disability or very poor performance (0–3) Poor performance (4–6) Moderate performance (7–9) Good performance (10–12)	- - - -	25 (19.2%) 37 (28.5%) 47 (36.2%) 21 (16.2%)
N = 108		
GPM *	28.81	±30.67
Total Fear of Falls FES-I score *	34.46 (±1.18)	
Older people with low concern *	16.92 (±2.24)	13 (12.0%)
Older people with moderate concern *	22.65 (±1.98)	20 (18.6%)
Older people with high concern *	40.65 (±7.93)	75 (69.4%)

Note: * Only valid cases (*n* = 108); ^£^ 6 missing values; ** 3 missing values.

**Table 3:** Relationship between physical performance measures and the FES-I, according to pain characteristics, Natal, RN, 2016.

	Balance Score (0–4) ^a^	Walking Speed (m/s) ^a^	Test Stand Up from Chair(s) ^a^	Total SPPB Score (0–12) ^a^	FES-I ^€^
**Pain**					
Yes	2.62 (±1.50)	0.63 (±0.29)	21.75 (±5.86)	5.89 (±2.74)	38.29 (±10.94)
No	2.85 (±1.36)	0.84 (±0.38)	18.58 (±6.51)	7.09 (±3.15)	29.50 (±10.59)
*p*-value	0.36	0.002 *	0.02 *	0.02 *	<0.001 **
**Acute Pain**					
Yes	2.29 (±1.57)	0.53 (±0.19)	23.92 (±3.96)	4.53 (±2.37)	37.31 (±11.83)
No	2.78 (±1.42)	0.75 (±0.35)	19.78 (±6.46)	6.69 (±2.95)	34.01 (±11.71)
*p*-value	0.19	0.04 *	0.01 *	0.005 *	**0.26**
**Chronic Pain**					
Yes	2.65 (±1.53)	0.65 (±0.31)	21.48 (±6.28)	6.06 (±2.80)	38.61 (±11.36)
No	2.77 (±1.38)	0.79 (±0.37)	19.31 (±6.29)	6.71 (3.11)	31.36 (±10.83)
*p*-value	0.66	0.02 *	**0.11**	**0.22**	0.001 **
GPM	−0.110	−0.324	0.308	−0.241	0.31 ^b^
*p*-value	0.22	0.001 *	0.004 *	0.007 *	0.001 ***^b^

^a^*t* test; ^b^ Spearman’s Correlation; * Statistical significance (*p* < 0.05); ** Statistical significance (*p* < 0.001); *** Statistical significance (*p* < 0.001); ^€^ only valid cases.

**Table 4:** Multivariate Analysis of Functional Performance, Natal, RN, 2016. (*n* = 133).

Variables	ß	95% CI	*p* Value
**Balance ^a^**			
Age	−0.045	−1.192: −0.78	0.01
Use of Hypnotics	−0.64	−1.19: −0.08	0.03
**Gait Speed ^b^**			
Age	−0.01	−0.02: −0.002	0.01
Acute pain	−0.23	−0.44: −0.02	0.03
**Chair Lift Test ^c^**			
Acute pain	3.7	−0.34: 7.75	0.04
**SPPB ^Total^**			
Age	−0.1	−0.16: −0.03	0.01
Acute pain	−1.9	−3.28: −0.43	0.01

^a^ Adjusted in the final model for sex, acute pain, hypnotic use, age, adjusted GPM and BMI; ^b^ Adjusted in the final model for sex, age, self-perception of health, MMSE, acute pain, chronic pain, pain distribution, pain intensity, GPM subscales and adjusted GPM; ^c^ Adjusted in the final model for sex, self-perception of health, age, Geriatric Depression Scale, acute pain, chronic pain, pain intensity, GPM subscales and adjusted GPM.

**Table 5:** Multivariate Analysis of the FES-I, Natal, RN, 2018. (*n* = 108).

Variables	ß	95% CI	*p*-Value
**Dichotomous Pain**			
Age	−0.19	−0.08: 0.46	0.17
Male	−4.60	−8.60: −0.60	0.02
No use of psychotropics	−4.70	−8.56: −0.85	0.01
Perceived good health	−0.89	−5.36: 3.57	0.69
GDS score	1.06	0.49: 1.63	<0.01
Dichotomous pain (yes)	5.47	1.51: 9.43	<0.01
**Categorical GPM**			
Age	0.19	−0.02: −0.002	0.18
Male	−5.06	−9.13: −1.00	0.01
No use of psychotropics	−4.40	−8.35: −0.44	0.03
Good health perceived	0.01	−4.92: 4.94	0.90
GDS score	1.05	0.46: 1.63	<0.01
**Pain Intensity (GPM)**			
Light	0	0	0
Moderate	4.04	−0.26: 8.36	0.06
Intense	7.15	0.27: 14.03	0.04
**Chronic Pain**			
Age	0.22	−0.05: −0.50	0.11
Male	−5.43	−9.43: −1.42	<0.01
No use of psychotropics	−4.79	−8.73: −0.85	0.01
Perceived health	−0.95	−5.61: 3.70	0.68
GDS score	1.04	0.46: 1.62	<0.01
Chronic pain (yes)	3.82	−0.22: 7.86	0.06

## Data Availability

All data generated during this study are included in this published article. The original datasets analyzed during the current study are available from the corresponding author on reasonable request.
